# Chemotherapy Enrichment of ID Family Expression Is Associated with IL-6 Signaling in Ovarian Cancer

**DOI:** 10.3390/cancers18081186

**Published:** 2026-04-08

**Authors:** Megan Anne Keene, Darren Lighter, Cassandra Brenner, Ixchel Urbano, Katelyn Shelby, Samuel F. Gilbert, Mikella Robinson, Carrie D. House

**Affiliations:** 1 Biology Department, San Diego State University, San Diego, CA 92106, USA; mkeene5367@sdsu.edu (M.A.K.);; 2 Moores Cancer Center, University of California San Diego, La Jolla, CA 92037, USA

**Keywords:** ovarian cancer, cancer stem-like cells, ID proteins, tumor microenvironment

## Abstract

**Background/Objectives**: Ovarian cancer (OC) remains the most lethal gynecologic malignancy, largely due to late-stage diagnosis and high rates of recurrence following platinum-based chemotherapy. Growing evidence implicates cancer stem-like cells (CSCs) in OC relapse, as these cells exhibit enhanced chemoresistance, stemness, epithelial–mesenchymal transition (EMT), and the capacity to remodel the tumor microenvironment. Inhibitors of DNA-binding (ID) 1-4 proteins are transcription factors with known redundancy; however, their collective role in OC chemotherapy response remains poorly defined. Here, we examined how ID family signaling responds to chemotherapy and contributes to CSC-associated features and microenvironment remodeling. **Methods**: Publicly available patient data, OC cell lines, and a subcutaneous xenograft mouse model were used to correlate changes in ID1-4 expression with CSCs, EMT, and the tumor microenvironment (TME). OC cell lines were used for in vitro assays to evaluate CSC features and IL-6 production in the presence of carboplatin and/or a small molecule inhibitor of ID proteins, AGX51. **Results**: Analysis of clinical datasets, cell lines, and in vivo models revealed enrichment of ID1-4 following chemotherapy, with additive increases across treatment cycles. In vivo ID2 and ID4 expression was associated with IL-6 secretion and loss of anti-tumoral macrophages. Pan-ID inhibition demonstrated that cumulative ID activity minimally supports CSC maintenance during chemotherapy, while more strongly regulating IL-6 secretion. **Conclusions**: IL-6 production from cancer cells was at least partially dependent on ID proteins, linking collective ID signaling to microenvironment remodeling and relapse potential in ovarian cancer.

## Simple Summary

Ovarian cancer relapse is driven in part by cancer stem-like cells (CSCs) and chemotherapy-enhanced tumor remodeling, yet the collective role of inhibitor of DNA-binding (ID) 1-4 proteins in this process remains unclear. Here, we show that ID1-4 are collectively enriched with chemotherapy in ovarian cancer models and patient samples, and this is associated with IL-6 expression and loss of anti-tumoral macrophages. While pan-ID inhibition minimally impacts CSC maintenance, it markedly suppresses IL-6 signaling, linking ID activity to chemotherapy-induced adaptations and relapse potential.

## 1. Introduction

Ovarian cancer (OC) remains the most lethal gynecologic malignancy in the United States. The largely nonspecific symptoms associated with advanced disease results in approximately 70% of patients being diagnosed at a late stage [1]. Consequently, the five-year survival rate for OC declines sharply from approximately 79.5% in early-stage disease to 17% in an advanced stage [2,3]. Even though most patients initially respond to platinum-based chemotherapy, over 80% experience chemoresistance and tumor recurrence within two years [4]. Emerging evidence suggests OC recurrence is driven, at least in part, by a small population of cancer stem-like cells, or CSCs, that exhibit enhanced tumor initiation capacity, chemoresistance, EMT plasticity, TME remodeling capabilities, and the ability to modulate macrophage polarization. CSCs exhibit multiple properties, including increased expression of stemness markers like SOX2, enhanced antioxidant aldehyde dehydrogenase (ALDH) activity, the capacity for spheroid formation, and can promote angiogenesis and immune evasion [1,5]. Because CSCs are difficult to target, identifying the key molecular regulators that govern these cells is essential for the development of improved diagnostic and therapeutic strategies aimed at preventing disease recurrence and improving long-term outcomes in ovarian cancer.

We previously showed that 3D spheroid culture, which enriches for CSCs, induces upregulation of SMAD1/5/8 signaling and inhibitor of DNA-binding (ID) family transcription factors [6]. ID proteins, known as both inhibitors of DNA-binding and as inhibitors of differentiation, comprise four members (ID1-4) of the basic helix-loop-helix (bHLH) family that lack a DNA-binding domain [7,8]. Acting as dominant-negative regulators of bHLH transcription factors, ID proteins are highly conserved and play critical roles in cell-intrinsic processes such as proliferation, differentiation, and survival, as well as in cell-extrinsic functions including angiogenesis and metastasis during both development and tissue homeostasis [7,8]. Although individual ID family members exhibit tissue- and stage-specific expression patterns during development, genetic deletion studies in mice reveal substantial functional redundancy among ID proteins in both developmental processes and tumorigenesis [9,10]. Whether similar redundancy exists among ID proteins in ovarian cancer and in response to chemotherapy remains unknown.

To our knowledge this is the first study to examine the cumulative behavior of ID1-4 in response to chemotherapy treatment and assess how this influences CSC-associated features and microenvironmental interactions linked to relapse. We first showed that analysis of clinical datasets from highly recurrent cancers revealed collective, cancer-agnostic ID expression patterns. In OC, *ID1-4* expression increased in cell lines and patient samples in response to chemotherapy treatment. We showed that ID family expression is increased with sequential exposure to chemotherapy; however, this response returned to baseline in vivo after tumor regrowth. Xenograft tumors with elevated ID expression exhibited increased IL-6 expression, no change in STAT3 or ERK phosphorylation, and decreased caspase 8 cleavage, which correlated with a loss of anti-tumoral, M1-like macrophages. Consistent with these findings, pharmacological pan-ID inhibition with small molecule, AGX51, only minimally perturbed *SOX2* expression, spheroid formation, and ALDH activity during chemotherapy treatment. In contrast, pan-ID inhibition significantly attenuated IL-6 secretion from OC cells. Together, these findings indicate ID proteins are enriched in chemoresistant cells and promote IL-6 secretion, which may induce microenvironment remodeling to increase relapse potential.

## 2. Materials and Methods

### 2.1. Cell Lines and Culture Conditions

OVCAR8, OVCAR4, CAOV4, and OV90 are cell lines derived from high grade serous ovarian cancer patients. OVCAR5 was derived from a patient with an advanced stage ovarian tumor with potential gastrointestinal origin. OVCAR8, OVCAR4, OVCAR5, and CAOV4 cells were obtained and authenticated from the NCI-Frederick DCTD tumor/cell line repository. OV90 was obtained and authenticated by ATCC: CRL-11732. All adherent cultures were maintained in standard RPMI media supplemented with 10% FBS and 1% 10,000 U/mL penicillin/streptomycin. Cells were tested for mycoplasma at least annually. Cells were maintained in culture for a maximum of 15 passages. Detailed cell line information as previously stated [5].

### 2.2. Public Database Analysis

The expression of *ID1-4* was compared across breast, colon, lung, pancreatic, and ovarian cancers and cell lines by accessing data on the cBio Cancer Genomics Portal Website [11,12] and reconstructing in excel by unique ID. Heatmap visualization of z-scores and k-means clustering of patients were performed using the ComplexHeatmap package (version 2.26.0 used with R, version 4.5.3) [13]. *ID1-4* expression for these same cancers across normal, tumoral, and metastatic tissue was compared using TNMplot (https://tnmplot.com/analysis/, accessed 5 December 2025) [14]. TNMplot is a web-based tool that calculates fold changes in normalized gene expression and applies a Kruskal-Wallis test, followed by a Dunn post hoc test, to compare normal, tumor, and metastatic samples. *ID1-4* expression across 17 datasets was evaluated for associations with overall survival using the web-based tool KMplotter (https://kmplot.com/analysis/index.php?p=service&cancer=ovar, accessed 6 August 2024), which applies univariate Cox proportional-hazards regression to generate Kaplan-Meier survival curves and calculate hazard ratios with corresponding confidence intervals [15]. Hazard ratios and confidence intervals were then reconstructed in GraphPad Prism (version 10.6.0). Pre- and post-neoadjuvant chemotherapy treatment (NACT) was obtained from https://doi.org/10.1158/0008-5472.CAN-21-1467 and reconstructed in excel by gene [16]. Pearson’s correlation analyses were performed using GraphPad Prism to assess correlations among gene expression levels within pre-NACT samples and within post-NACT samples.

### 2.3. Gene Expression by qRT-PCR

RNA was collected, converted to cDNA, and analyzed by qRT-PCR as previously stated [5]. Briefly, 200,000–400,000 cells were plated in 10 cm plates overnight and then treated with 20 μM AGX51 (MedChemExpress, Monmouth Junction, NJ, USA) for 24 h, followed by 72 h of vehicle or carboplatin (40 or 100 μM), in the presence or absence of AGX51. RNA was collected using the MacheryNagel RNA Kit (Takara Bio USA, San Jose, CA, USA) and converted to cDNA using the High-Capacity cDNA Reverse Transcription Kit (Thermo Fisher Scientific, Waltham, MA, USA). Quantitation of gene expression was performed with Taqman Fast Advanced Master Mix and TaqMan probe assays (Thermo Fisher Scientific, Waltham, MA, USA) (Table 1). Gene expression was normalized to *GAPDH* as a control using the comparative threshold cycle method. 

### 2.4. Lysate Preparation and Western Immunoblotting

Whole cell protein was extracted and analyzed as previously stated in [5]. Samples were collected using RIPA lysis buffer (Thermo Fisher Scientific, Waltham, MA, USA) supplemented with the Halt Protease and Phosphatase Inhibitor Cocktail, quantified by Pierce Rapid Gold BCA Protein Assay Kit, run on SDS-PAGE using NuPAGE 4–12% Bis-Tris Protein Gels and NuPAGE MOPS SDS Running Buffer (Invitrogen, Carlsbad, CA, USA), and transferred to PVDF Transfer Membrane, blocked with 5% milk, and incubated overnight at 4 °C with primary antibodies. Secondary antibodies were incubated at room temperature for 1 h (anti-rabbit IgG HRP-linked (1:2000) and anti-mouse IgG, HRP-linked (1:2000) (both from Cell Signaling Technology, Danvers, MA, USA)). GAPDH (Millipore, Burlington, MA, USA, 1:6000) was used to normalize the data. SuperSignal or Femto Chemiluminescent Substrate (both from Thermo Fisher Scientific, Waltham, MA, USA) was used for protein detection in the iBright CL1000 Imaging System (Thermo Fisher Scientific, Waltham, MA, USA). Membranes were stripped and reprobed for each ID protein and GAPDH using the OneMinute Western blot Stripping kit (GM Biosciences, Rockville, MD, USA). iBright Analysis Software (Invitrogen, Carlsbad, CA, USA) was used to determine relative protein expression. For AGX51 treatment, primary antibodies used include ID1 (BCH-1/195-14, BioCheck, Foster City, CA, USA, 1:2000), ID2 (D39E8, Cell Signaling, Danvers, MA, USA, 1:1000), ID3 (D16D10, Cell Signaling, Danvers, MA, USA, 1:1000), and ID4 (sc-365656, Santa Cruz Biotechnology, Dallas, TX, USA, 1:200). For tumor lysates, primary antibodies include P-STAT3 (9145, Cell Signaling Technology, Danvers, MA, USA, 1:1000), T-STAT3 (4904, Cell Signaling Technology, Danvers, MA, USA, 1:1000), P-ERK (4377, Cell Signaling Technology, Danvers, MA, USA, 1:2000), T-ERK (9102, Cell Signaling Technology, Danvers, MA, USA, 1:2000), SOX2 (2748, Cell Signaling Technology, Danvers, MA, USA, 1:1000), caspase 8 (MA1-41280, Thermo Fisher, Waltham, MA, USA, 1:1000), and RIP (3493, Cell Signaling Technology, Danvers, MA, USA, 1:1000). Original western blots are presented in File S1. 

### 2.5. Animal Experiments

Animal studies were performed as previously stated [17]. Animal studies were approved by the SDSU Animal Care and Use Committee (protocol approval numbers 18-04-006H). For subcutaneous xenografts, 500,000 OV90 cells in 1:1 Matrigel in PBS were subcutaneously injected into the left flank of 8-week-old female athymic Nu/Nu mice. Power analysis indicates using 4 to 6 mice/group achieves a conservative effect size of 0.4. Mice were weighed and tumors were measured with blinded groups twice weekly. Once tumors reached 150 mm^3^, mice were randomized and treated with either vehicle or carboplatin (50 mg/kg) delivered via intraperitoneal injection once per week for 3 weeks. To evaluate progressive changes, mice were sacrificed in two groups after the third dose of carboplatin: <4 days (residual) or >4 days (regrown) until tumors reached 20 mm in length or after 90 days of growth. Mice tumor tissues were collected and analyzed for protein analysis and for macrophage markers CD80 and CD163 by flow cytometry.

### 2.6. Immunohistochemistry (IHC)

IHC was performed as previously stated [17]. Tumors were fixed in 10% neutral buffered formalin, paraffin-embedded, and sectioned at 5 μm. Following citrate-based antigen retrieval and quenching of endogenous peroxidase activity, sections were incubated with primary antibodies overnight at 4 °C for ID1 (BCH-1/195-14, BioCheck, Foster City, CA, USA, 1:2000), ID2 (BCH-3/9-2-8, BioCheck, Foster City, CA, USA, 1:2000), ID3 (sc-56712, Santa Cruz Biotechnology, Dallas, TX, USA, 1:200), and ID4 (sc-365656, Santa Cruz Biotechnology, Dallas, TX, USA, 1:200). HRP-conjugated secondary antibodies for 1 h at room temperature and visualized with DAB. Four blinded, randomly selected fields per section were imaged using an Olympus BX51 microscope (EvidentScientific, Tokyo, Japan), and DAB staining was quantified using ImageJ with the FIJI color deconvolution plugin (version 2.3.0).

### 2.7. Spheroid Assays

Spheroid formation was performed as previously stated [5]. For spheroid formation, 500 cells per well were seeded into ultra-low attachment (ULA), flat-bottom 96-well plates in normal culture medium. For conditions with pretreatment of AGX51, 10,000 cells were seeded in 12-well plates overnight and exposed to AGX51 for 20 μM for 48 h prior to seeding for spheroid formation, then cells were grown overnight in ULA plates before continuing treatment with either AGX51 (20 μM), carboplatin (40 or 100 μM), or in combination, as stated in the figure legends. For spheroid dissemination, spheroids were grown in stem cell media and treated with AGX51 and/or carboplatin after 5 days of growth. Stem cell media consists of DMEM:F12 supplemented with 1% KnockOut serum replacement, 0.4% bovine serum albumin, and 0.1% insulin-transferrin-selenium and supplemented with human recombinant epidermal growth factor (EGF) and basic fibroblast growth factor (FGF) every 2-3 days for final concentration of 20 ng/mL and 10 ng/mL, respectively. For all spheroid assays, after 4-7 days of incubation, spheroids were stained with Hoechst dye and imaged at 10× magnification using the ImageXpress Pico (Molecular Devices, San Jose, CA, USA) system. Spheroid diameters ranging from 30 to 500 μm for formation or 80 to 500 μm for dissemination were quantified and analyzed using CellReporterXpress software (version 2.9.19394, Molecular Devices, San Jose, CA, USA).

### 2.8. Chemosensitivity Assays

Viability assay was performed as previously stated [5]. A total of 1000 cells were seeded into 96-well, white clear-bottom plates as stated in the figure legends and viability was assessed with 2.0 CellTiter-Glo luminescent reagent, according to manufacturer’s instructions on a Varioskan plate reader. For Supplemental Figure S3A, cells were treated with either AGX51 (40 μM), carboplatin (40 or 100 μM), or in combination. For Supplemental Figure S3B, cells were pretreated with AGX51 (40 μM) for 24 h, followed by drug washout and subsequent vehicle or carboplatin treatment (40 or 100 μM) for 72 h. For Supplemental Figure S3C, vehicle or AGX51 (20 μM) was introduced for 24 h followed by 72 h of vehicle or carboplatin (40 or 100 μM), in the presence or absence of AGX51.

### 2.9. Flow Cytometry

Flow cytometry experiments were completed as previously described [18]. Briefly, 10,000 cells were seeded in 12-well plates overnight and exposed to AGX51 for 24 h prior to seeding for flow cytometry. After 24 h, cells were treated with either AGX51 (20 or 40 μM), carboplatin (40 or 100 μM), or in combination, as stated in the figure legends. Cells were collected with cell stripper and stained for ALDH activity using the AldeFluor assay kit (Stemcell Technologies, Vancouver, BC, USA) per the manufacturer’s instructions. Flow cytometry was performed on a BD LSRII.

### 2.10. Enzyme-Linked Immunosorbent Assay (ELISA)

The concentration of IL-6 was assessed using DuoSet sandwich ELISA (R&D Systems, Minneapolis, MN, USA). Conditioned media samples were collected from the flow cytometry experiments described above, prepared, and analyzed per the manufacturer’s instructions. Samples were normalized to their viable cell quantification.

### 2.11. siRNA Transfections

Knockdown studies were completed as done previously [5]. A total of 100,000 cells were plated in a six-well overnight. The following day, 30 nM pools of 4 siRNAs targeting either ID3 or ID4 or a non-targeting control (Dharmacon, Lafayette, CO, USA) were transfected using Lipofectamine RNAiMAX (Invitrogen, Carlsbad, CA, USA) per manufacturer’s instructions. Cells were allowed 48 h before being collected for downstream experiments. Cells were replated and, after 24 h, treated with vehicle or carboplatin (40 or 100 μM) for 72 h, and collected for viability assay, gene expression by qRT-PCR, or conditioned media for ELISA, as stated in figure legends.

### 2.12. Statistical Analysis

Statistics were generated using Prism 10 with data acquired from at least three independent biological replicates. Results are presented as mean ± SEM, unless otherwise stated in the figure legends. Outliers were identified using the Grubbs’ test. Significance was calculated using either an unpaired *t*-test for comparisons of two means or ANOVA for comparisons of three or more means with a post hoc test to identify differences between groups as described in figure legends. Pearson’s correlation coefficient was used to assess the linear relationship between pairs of gene expression variables. Differences between means are considered statistically significant at the 95% level (*p* < 0.05). The lack of an asterisk or ns indicates non significance.

## 3. Results

### 3.1. Patient-Level Patterns of ID1-4 Expression Across Several Prevalent Cancers

**Figure 1:**
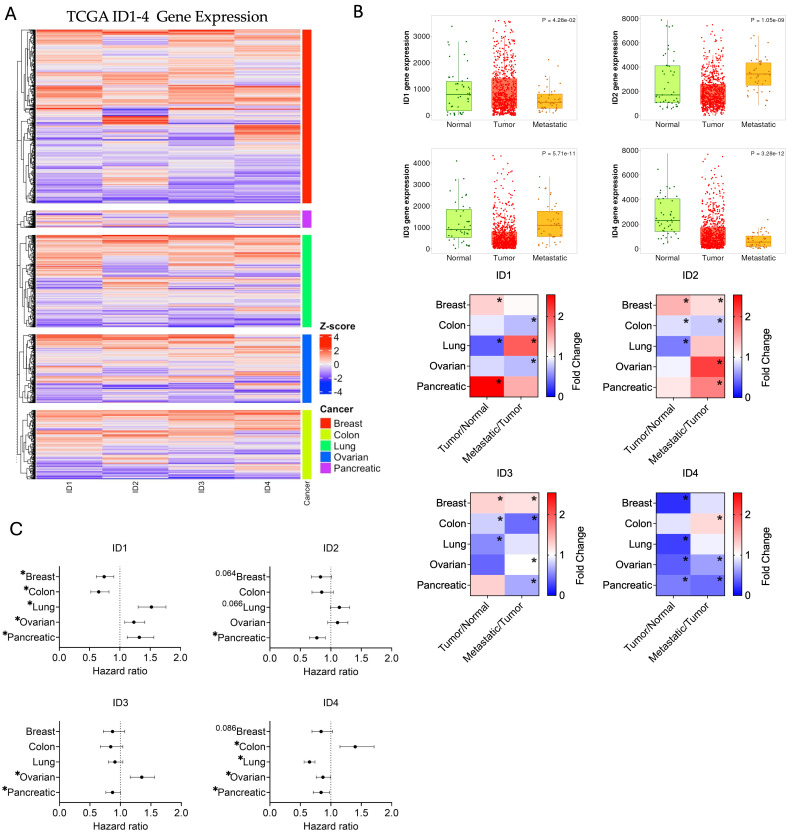
Patient-level patterns of *ID1-4* expression across several prevalent cancers. (**A**) Heatmap of z-scores for *ID1-4* gene expression with k-means clustering of breast, colon, lung, ovarian, and pancreatic cancers. (**B**) Representative TNMplots for *ID1-4* gene expression for ovarian cancer (top) and heatmap of *ID1-4* gene expression fold change (bottom) between tumor vs. normal tissue and metastatic vs. tumor tissue in breast, colon, lung, ovarian, and pancreatic cancers. Kruskal-Wallis test, Dunn post hoc test. Example TNMplots of ovarian cancer. (**C**) Hazard ratios of *ID1-4* gene expression for overall survival in breast, colon, lung, ovarian, and pancreatic cancers generated with KMplotter. * *p* < 0.05.

To explore the collective role and potential redundancy of *ID1-4* across multiple cancer types, we first analyzed TCGA gene expression datasets across several highly recurrent malignancies, including breast, colon, lung, ovarian, and pancreatic cancer (Figure 1A). Because previous studies have largely examined overall patient trends in *ID1-4* expression in relation to copy number alterations, mutations, and methylation status [19], we applied k-means clustering of *ID1-4* z-scores normalized expression to investigate patient-level patterns of coordinated ID expression across these cancers. Heatmap analysis revealed substantial heterogeneity in *ID1-4* expression across cancer subtypes, with no single cancer type uniformly exhibiting higher or lower ID expression. Instead, each cancer displayed distinct subsets of patients with either high or low ID expression. Notably, individual ID family members tended to exhibit concordant expression patterns within individual patients, with coordinated high or low expression across multiple ID family genes, suggesting pan regulation of ID1-4. To determine if these patterns were associated with tumor progression, we utilized the web-tool TNMplot to compare *ID1-4* expression in normal tissue, primary tumors, and metastatic tumors. Fold-change analysis from normal to tumor and from tumor to metastatic revealed similar trends between ID family members within each cancer type (Figure 1B). For example, breast cancer exhibited a progressive increase in *ID1-3* expression from normal tissue to primary tumors to metastatic tumors, whereas ovarian (*ID1*, *3-4*) and colon (*ID1-3*) cancers exhibited decreased expression across these stages. To investigate the association of *ID1-4* gene expression with clinical outcomes, we utilized web-tool KMplotter to evaluate the overall survival of patients stratified by high versus low *ID1-4* expression. Univariate Cox proportional-hazards regression revealed cancer-specific prognostic patterns, with *ID1-3* collectively associated with overall survival for OC patients, and *ID1-2*, and *ID4* predictive for lung cancer (Figure 1C). Together, these findings suggest a model of functional redundancy among ID family members in recurrent cancers, with ID1-3 potentially playing more overlapping roles in tumor progression and treatment response in ovarian cancer.

### 3.2. Chemotherapy-Associated Upregulation of ID Expression in Ovarian Cancer

**Figure 2:**
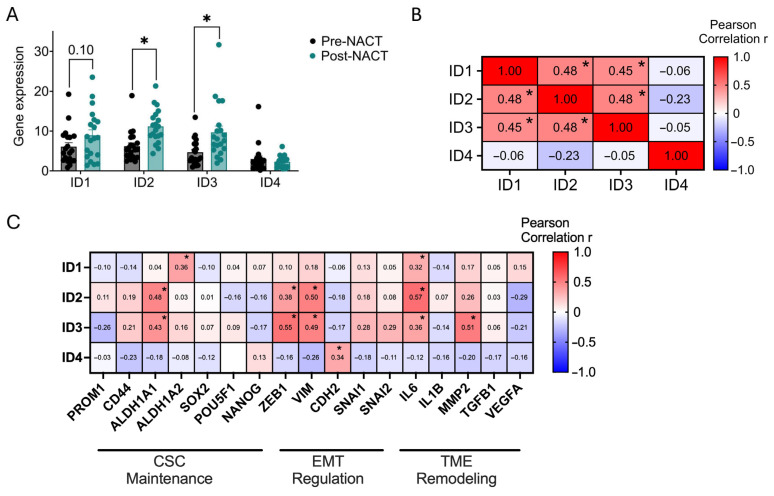
Chemotherapy-associated upregulation of ID expression in ovarian cancer models. (**A**) *ID1-4* gene expression from pre- and post-treatment ovarian cancer tumors (Javellana et al., *n* = 20), paired *t*-test. (**B**) Pearson’s correlation of *ID1-4* gene expression in ovarian cancer tumors (Javellana et al., *n* = 20). (**C**) Pearson’s correlation of *ID1-4* genes expression with selected CSC, EMT, and TME genes in patient tumors (Javellana et al., *n* = 20). * *p* < 0.05.

Interestingly, in ovarian cancer, *ID1* and *ID3* expression decreased with disease progression yet remained associated with poorer clinical outcomes (Figure 1); therefore, we next investigated whether ID expression is altered in ovarian cancer clinical samples following chemotherapy treatment. Gene expression analysis of paired tumor samples collected before and after neoadjuvant chemotherapy treatment (NACT) revealed significant increases in *ID2* and *ID3* gene expression, with *ID1* expression also trending upward, while *ID4* expression remained largely unchanged (Figure 2A). Analysis of ID gene expression correlations in paired OC tumor samples collected before and after NACT highlighted significant correlations among *ID1*, *ID2*, and *ID3* but not *ID4* (Figure 2B). To identify the signaling pathways that ID expression potentially regulates during chemotherapy treatment, we performed a literature-based curation of ID-associated signaling networks reported for individual family members and grouped these pathways into three functional categories: CSC maintenance, EMT regulation, and TME remodeling (Figure 2C). Using patient samples shown in Figure 2A, we performed correlation analyses between *ID1-4* and representative genes from each category. CSC-associated genes included *PROM1*, *CD44*, *ALDH1A1*, *ALDH1A2*, *SOX2*, *POU5F1*, and *NANOG* [20,21,22]. EMT-associated genes included *ZEB1*, *VIM*, *CDH2*, *SNAI1*, and *SNAI2* [23,24,25]. TME remodeling factors included *IL6*, *IL1B*, *MMP2*, *TGFB1*, and *VEGFA* [26,27]. Correlation analysis revealed significant correlations between *ID2* and *ID3*, and to a lesser extent *ID1*, to genes across all three functional categories, including *ALDH1A1*, *ALDH1A2*, *ZEB1*, *VIM*, *CDH2*, *IL6*, and *MMP2*. Collectively, this data suggests that ID expression may increase in response to chemotherapy and may influence CSC-associated features like CSC maintenance, EMT regulation, and TME remodeling.

### 3.3. Chemotherapy-Associated Upregulation of ID Expression Is Cycle-Dependent

**Figure 3:**
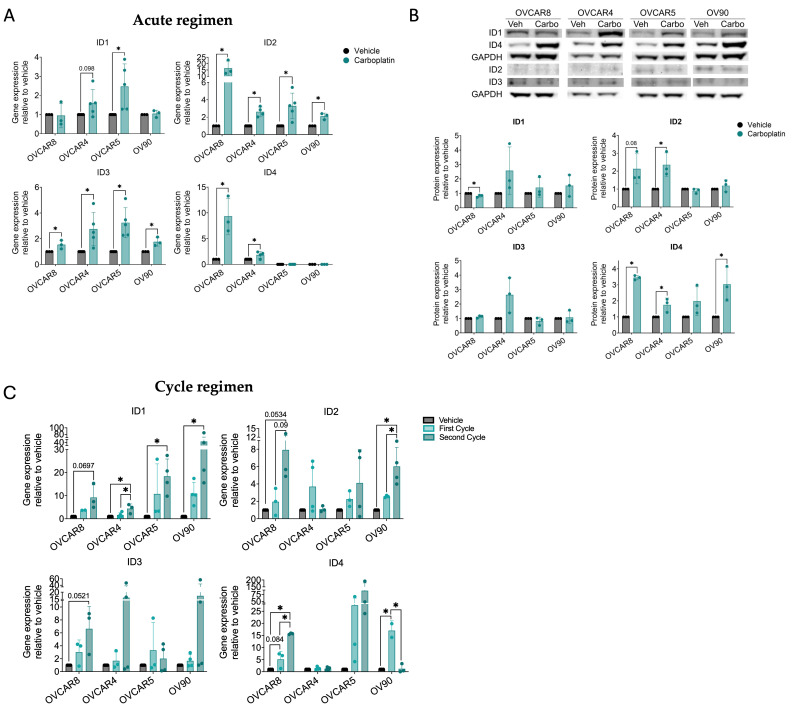
Chemotherapy-associated upregulation of ID expression is cycle-dependent. (**A**) Relative *ID1-4* gene expression in OVCAR8, OVCAR4, OVCAR5, and OV90 cells treated with vehicle or IC50 carboplatin for 72 h (125 μM for OV90 and OVCAR5, 62.5 μM for OVCAR8 and OVCAR4). *n* = 3–5, unpaired *t*-test. (**B**) Relative ID1-4 protein expression of OVCAR8, OVCAR4, OVCAR5, and OV90 cells treated with vehicle or carboplatin for 72 h. *n* = 3, unpaired *t*-test. (**C**) Relative *ID1-4* gene expression of OVCAR8, OVCAR4, OVCAR5, and OV90 cells that underwent vehicle, one cycle, or two cycles of carboplatin treatment at IC30 (20 μM for OVCAR8 and OVCAR4, 30 μM for OVCAR5, and 54 μM for OV90). *n* = 3–4, one-way ANOVA, Tukey post hoc test. * *p* < 0.05.

To assess ID expression changes in vitro, we utilized four cell lines (OVCAR8, OVCAR4, OVCAR5, and OV90), selected to reflect the heterogeneity of baseline *ID1-4* observed in clinical samples. Interrogation of CCLE dataset indicated that OVCAR4 typically exhibits ID expression in the highest quartile, whereas OV90 often falls within the lowest quartile (Supplemental Figure S1A). Based on baseline gene expression profiles, the cell lines were stratified into two groups: those exhibiting high baseline expression of two or more ID genes greater than 0.1 normalized gene expression (OVCAR4 expressing *ID1-4* and OVCAR8 expressing *ID1* and *ID3*) and those with predominant baseline expression of a single ID gene (OVCAR5 and OV90 expressing *ID2*) (Supplementary Figure S1B). To evaluate acute responses, ID expression was examined following 72 h of carboplatin treatment (“acute regimen”). *ID2* and *ID3* were consistently and significantly upregulated across most cell lines at the mRNA level (Figure 3A). Although *ID1* and *ID4* exhibited greater variability among cell lines, they followed a similar overall pattern of chemotherapy-enhanced expression. At the protein level, expression was more heterogenous, with significant increases in ID4 expression across most cell lines and variable increases in ID1 and ID2 expression (Figure 3B). Overall, ID1 and ID4 protein expression levels were higher across OC cell lines, whereas ID2 and ID3 were more weakly expressed. This variable abundance may reflect ID family post-translational regulation, such as proteasomal degradation, or expression within a small subpopulation of cells such as CSCs. This data suggests that expression of some ID proteins is preserved following chemotherapy exposure.

We next examined whether ID expression is sustained after multiple cycles of chemotherapy, which would enrich for the most drug-resistant cells including CSCs [28,29]. Using a previously established in vitro treatment protocol that models clinical carboplatin regimens, cells were subjected to two cycles of IC30 carboplatin treatment with drug washout and recovery periods between cycles to mimic the biological half-life of chemotherapy (“cycle regimen”) [5] (Figure 3C). We observed a progressive increase in ID expression with successive treatment cycles. Specifically, *ID1* expression increased significantly following each carboplatin exposure across all cell lines, while *ID2* and *ID3* were significantly increased in most cell lines, and *ID4* exhibited a similar trend but was restricted to OVCAR8 and OVCAR5 cells. Notably, cell lines with lower baseline ID expression (OVCAR8, OVCAR5, and OV90) showed more robust enrichment of ID expression following repeated chemotherapy, whereas the highest baseline cell line (OVCAR4) primarily exhibited increases in *ID1* and *ID3*. Furthermore, although *ID4* expression in OVCAR5 and OV90 cells was below the detection threshold in the acute treatment regimen, *ID4* became detectable after a single cycle in the cycle regimen and by Western blot analysis. This pattern suggests expression within a small cellular subpopulation and/or enhanced protein stability mediated by post-translational regulation, consistent with observations by others following chemotherapy [30]. Taken together, these data suggest that repeated carboplatin treatment drives ID expression and/or enriches for cells with high ID expression.

### 3.4. Chemotherapy-Enriched ID Expression Is Time Point-Dependent and May Promote a Pro-Tumoral Macrophage Phenotype in a Xenograft Mouse Model

**Figure 4:**
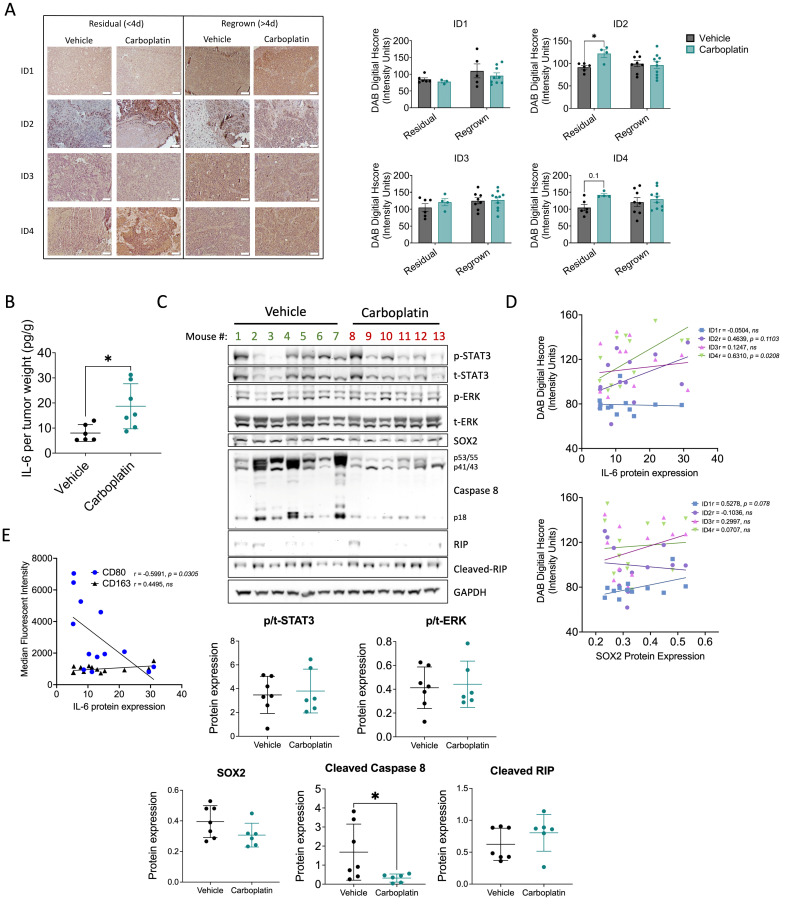
Chemotherapy-enriched ID expression is time point-dependent and may promote a pro-tumoral macrophage phenotype in a xenograft mouse model. (**A**) ID1-4 IHC of OV90 subcutaneous xenograft tumor mouse model, collected from residual (<4 days after three cycles of carboplatin) and regrown (>4 days after three cycles of carboplatin) tumors, *n* = 3–10. Each dot represents one mouse. In representative images, white bar indicates 100 μm. Two-way ANOVA, Sidak post hoc test. (**B**) IL-6 concentration relative to tumor weight. *n* = 6–7, unpaired *t*-test. (**C**) Expression of p-STAT3, t-STAT3, p-ERK, t-ERK, SOX2, caspase 8, RIP, and GAPDH proteins in residual tumors collected vehicle and carboplatin-treated mice. Quantification of p/t-STAT3, p/t-ERK, SOX2, cleaved caspase 8, and cleaved RIP normalized to GAPDH. *n* = 6–7, unpaired *t*-test. (**D**) Pearson’s correlation of ID1-4 digital Hscore and IL-6 (**top**) or SOX2 (**bottom**) protein expression from OV90 subcutaneous xenograft tumors described above. (**E**) Pearson’s correlation of median fluorescent intensity of CD80 and CD163 expression by flow cytometry and IL-6 protein expression from OV90 subcutaneous xenograft tumors described above. * *p* < 0.05.

To determine the durability of chemotherapy-enriched ID expression, we analyzed OV90 subcutaneous xenograft tumor samples collected either immediately following chemotherapy (“residual”, <4 days after three cycles of carboplatin) and at regrowth (“regrown”, >4 days after three cycles of carboplatin) [17]. ID2 and ID4 protein levels were transiently elevated in residual tumors but returned to baseline at later time points (Figure 4A), consistent with CSCs that are enriched at residual time points. To evaluate the in vivo relevance of factors associated with the temporal induction of ID2 and ID4 that may influence CSC maintenance, EMT regulation, and TME remodeling, we focused our analysis on IL-6, a cytokine known to support CSC-associated features [28], EMT-related processes [31], and TME plasticity [32]. We analyzed IL-6 production in response to chemotherapy in residual tumors described above. Tumors collected following three cycles of carboplatin treatment showed significantly increased IL-6 expression compared with mice receiving vehicle treatment (Figure 4B). To assess the action of IL-6 and explore additional ID-related pathways, we next quantified STAT3 and ERK phosphorylation, SOX2 expression, and cleavage of caspase 8 and RIP. Residual tumors exhibited decreased caspase 8 cleavage with persistent STAT3 and ERK phosphorylation, SOX2 expression, and RIP cleavage relative to vehicle treatment (Figure 4C), suggesting reduced apoptotic OC cell death and persistent STAT3 signaling. Interestingly, IL-6 expression correlated significantly with ID4 and trended with ID2 expression but not SOX2 expression (Figure 4D). Since IL-6 has been linked to the induction of pro-tumoral, M2-like macrophage phenotypes in OC [33], we next examined associations between IL-6 expression and macrophage populations, using flow cytometry data of CD80 and CD163 macrophage surface markers that was previously reported by our lab [34]. Single cells were isolated from tumors and analyzed for M1-like (CD80) and M2-like (CD163) expression by flow cytometry. Increased IL-6 expression was negatively correlated with median fluorescence intensity (MFI) of the anti-tumoral, M1-associated marker CD80, while expression of the pro-tumoral, M2-associated marker CD163 was maintained (Figure 4E). In support of ID proteins promoting IL-6-associated TME remodeling rather than acting directly on CSC or EMT features, as seen in our in vivo model, IL-6 treatment did not significantly alter cell viability or the expression of *SOX2*, *NIK*, or *VIM* over 72 h (Supplemental Figure S2A,B), nor did prolonged IL-6 exposure affect spheroid formation (Supplemental Figure S2C). These findings suggest that increased IL-6 expression in ID-high tumors may suppress anti-tumoral macrophage activity and promote a more permissive TME.

### 3.5. Pan-ID Inhibition Partially Attenuates Chemotherapy-Enhanced CSC Features

**Figure 5:**
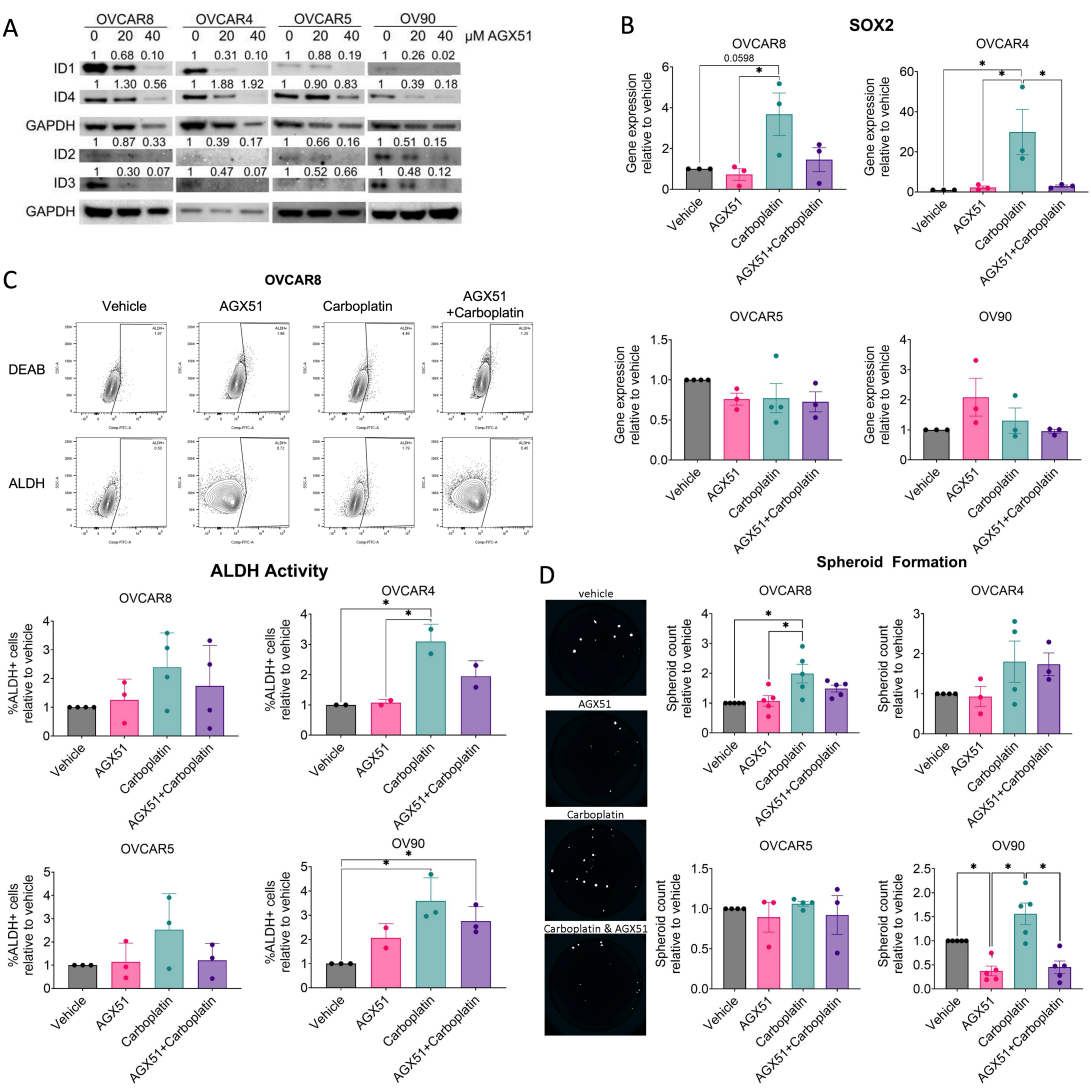
Pan-ID inhibition partially attenuates chemotherapy-enhanced CSC features. (**A**) ID1-4 protein expression of OVCAR8, OVCAR4, OVCAR5, and OV90 cells treated with vehicle, 20, or 40 μM AGX51 for 48 h. Representative Western blot (*n* = 3–4). (**B**) Relative *SOX2* gene expression of OVCAR8, OVCAR4, OVCAR5, and OV90 cells pretreated with vehicle or 20 μM AGX51 for 24 h, then continued concurrently with vehicle or 100 μM carboplatin (40 μM for OVCAR8) treatment for 72 h. *n* = 3–4, one-way ANOVA, Tukey post hoc test. (**C**) Relative activity of ALDH by flow cytometry in OVCAR8, OVCAR4, OVCAR5, and OV90 cells pretreated with vehicle or 20 μM AGX51 for 24 h, then continued concurrently with vehicle or 100 μM carboplatin (40 μM for OVCAR8) treatment for 72 h. *n* = 3–4, one-way ANOVA, Tukey post hoc test. (**D**) Relative spheroid formation of OVCAR8, OVCAR4, OVCAR5, and OV90 cells with 48 h pretreatment of vehicle or 20 μM AGX51, 24 h spheroid induction, and 72 h treatment of vehicle, 20 μM AGX51 and/or 100 μM carboplatin (40 μM for OVCAR8). Representative spheroid images of OVCAR8. * *p* < 0.05.

To evaluate the cumulative effects of ID proteins in vitro, we utilized AGX51, a recently identified small molecule pan-ID inhibitor [35]. Using a 48 h time point, treatment with 20 or 40 μM AGX51 reduced ID1-4 expression in most cases across OVCAR8, OVCAR4, OVCAR5, and OV90 cells (Figure 5A). Previous studies have shown that 40 μM or higher concentrations of AGX51 lead to reactive oxygen species accumulation and loss of cell viability, effects that are attributed to off-target toxicity rather than specific pan-ID inhibition [35]. Consistent with this, treatment with 40 μM AGX51 for 48 or 72 h resulted in significant loss of viability, both alone and in combination with carboplatin, compared with carboplatin treatment alone (Supplemental Figure S3A). To reduce off-target toxicity we pretreated for 24 h with 40 μM AGX51, followed by drug washout and subsequent carboplatin treatment for 72 h; however, this regimen again caused substantial loss of viability (Supplemental Figure S3B). Therefore, we administered 20 μM AGX51 as a pretreatment which was maintained in the media during subsequent carboplatin treatment to reduce AGX51-induced cytotoxicity while maximizing ID1-4 knockdown (Supplemental Figure S3C). Notably with this dosing strategy, AGX51 did not significantly increase sensitivity to carboplatin treatment, suggesting ID proteins may not mediate chemoresistance. Therefore, pretreatment at 20 μM AGX51 was chosen for subsequent experiments in order to reduce off-target toxicity by using a reduced concentration over an extended exposure period and to suppress chemotherapy-induced upregulation of ID proteins. Furthermore, this dosing strategy allowed modeling of a clinically feasible regimen in which ID-dependent responses are attenuated during chemotherapy.

To further evaluate the pathways involved in CSC maintenance and EMT regulation during chemotherapy, we next examined the dependence on ID proteins for the candidate genes identified in patient samples evaluated in Figure 2C. Factors that were significantly correlated with ID expression, including *MMP2*, *ZEB1*, *VIM1*, and *CDH2*, were neither significantly enriched with carboplatin, with the exception of *VIM1* in OVCAR4, nor responsive to pan-ID inhibition with AGX51 (Supplemental Figure S4A). We next examined established ovarian CSC-associated factors *SOX2* and *NOTCH3* [5,36]. *NOTCH3* expression was not altered by chemotherapy nor by AGX51 treatment (Supplemental Figure S4B). In contrast, although *SOX2* expression did not correlate with *ID1-4* in Figure 2C, carboplatin treatment significantly increased *SOX2* expression, which was abrogated by AGX51 treatment (Figure 5B). This ID-mediated *SOX2* enrichment was observed in cell lines with higher baseline ID expression (OVCAR8 and OVCAR4), but not in cell lines with lower baseline ID expression (Supplemental Figure S1B). Furthermore, OVCAR5 and OV90 exhibited higher baseline *SOX2* levels (Supplemental Figure S4C), which may limit the inducibility of *SOX2* [5].

Given the ID-mediated regulation of *SOX2*, we next assessed ALDH activity, which was significantly correlated with ID1-4 expression (Figure 2C) and is an established CSC marker associated with high SOX2 expression [37]. As previously reported [6], ALDH activity increased following carboplatin treatment across all cell lines, reaching significance in OVCAR4 and OV90 cell lines (Figure 5C). While ALDH activity was partially attenuated by pan-ID inhibition, this effect did not reach significance in any cell lines. To further assess functional significance of ID1-4, we evaluated spheroid formation, which we and others have shown to be regulated by SOX2 and ALDH activity [5,37]. Consistent with *SOX2* enrichment, cell lines that exhibited ID-mediated *SOX2* upregulation (OVCAR8 and OVCAR4) also showed increased spheroid formation following carboplatin treatment (Figure 5D). However, this was not significantly reduced with AGX51, with the exception of OV90 (Figure 5D). In contrast, established spheroids from OVCAR8 and OVCAR4 cells cultured for several days before treatment exhibited limited responsiveness to carboplatin and none of the spheroids were responsive to AGX51 (Supplemental Figure S4D). Collectively, these data suggest that CSC-associated responses to chemotherapy are minimally dependent on pan-ID signaling.

### 3.6. Pan-ID Inhibition Attenuates Chemotherapy-Enhanced IL-6 Secretion

**Figure 6:**
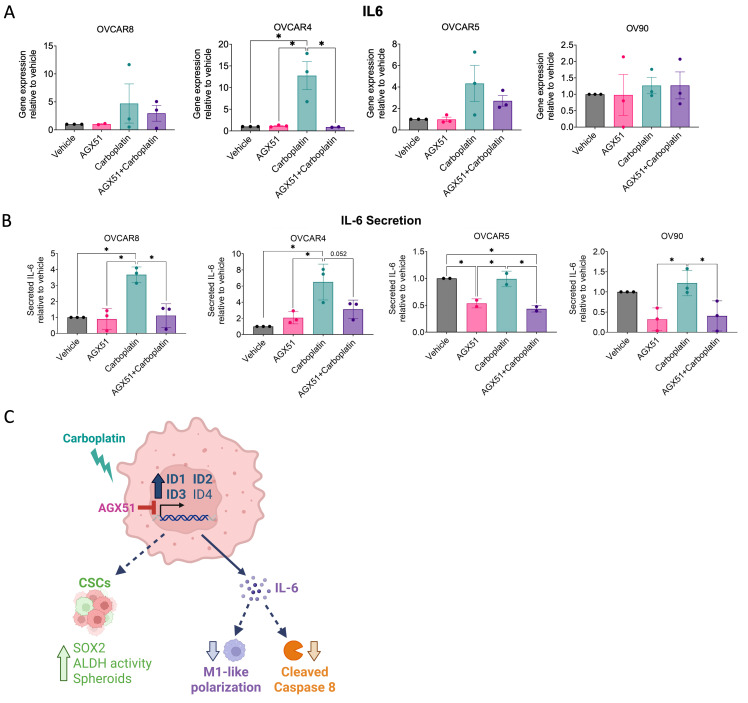
Pan-ID inhibition attenuates chemotherapy-enhanced IL-6 secretion. (**A**) Relative *IL6* gene expression of OVCAR8, OVCAR4, OVCAR5, and OV90 cells pretreated with vehicle or 20 μM AGX51 for 24 h, then continued concurrently with vehicle or 100 μM carboplatin (40 μM for OVCAR8) treatment for 72 h. *n* = 3, one-way ANOVA, Tukey post hoc test. (**B**) Relative IL-6 secretion in conditioned media from OVCAR8, OVCAR4, OVCAR5, and OV90 cells pretreated with vehicle or 20 μM AGX51 for 24 h, then continued concurrently with vehicle or 100 μM carboplatin (40 μM for OVCAR8) treatment for 72 h. *n* = 3, one-way ANOVA, Tukey post hoc test. (**C**) Summary schematic. Chemotherapy-associated cumulative ID induction significantly enhances IL-6 expression (solid black arrow) and minimally supports CSC features (dotted black arrow), potentially contributing to tumor plasticity and relapse potential. * *p* < 0.05.

Next, we investigated IL-6, a cytokine previously implicated in our in vivo model (Figure 4), that correlated with ID expression in Figure 2C, and has been associated with ovarian CSCs, EMT, and TME remodeling [28]. At the transcript level, *IL6* expression increased in response to carboplatin treatment in three cell lines (OVCAR8, OVCAR4, and OVCAR5) and was partially attenuated by pan-ID inhibition with AGX51 (Figure 6A). To determine whether these transcriptional changes translated into altered cytokine production, conditioned media was collected, normalized to cell viability, and analyzed for secreted IL-6. Compellingly, IL-6 secretion was significantly attenuated by AGX51 treatment in all cell lines (Figure 6B). In cell lines with higher baseline ID expression (OVCAR8 and OVCAR4), carboplatin treatment induced robust IL-6 secretion, which was attenuated by AGX51. In contrast, cell lines with lower baseline ID expression (OVCAR5 and OV90) did not increase IL-6 secretion following carboplatin treatment but still exhibited reduced IL-6 secretion in response to AGX51 treatment, with and without carboplatin treatment. Notably, these cell lines express only *ID2* at baseline, a family member previously linked to IL-6 regulation in prostate cancer [38]. Cells with higher baseline ID expression (OVCAR8 and OVCAR4) exhibited increased baseline *IL6* gene expression and IL-6 secretion, suggesting baseline IL-6 regulation by other pathways (Supplemental Figure S5A,B). Taken together, these data suggest that ID family activity contributes to OC cell-derived IL-6 expression that may be associated with TME remodeling.

To confirm that the effects of AGX51 on IL-6 expression following chemotherapy were not due to off-target activity and to clarify the roles of individual ID family members in regulating this response, we employed siRNA-mediated knockdown of ID proteins during chemotherapy treatment and assessed cell viability, knockdown efficiency, and IL-6 secretion. Because ID1 and ID2 have previously been linked to IL-6 expression [27,39], potentially confounding chemotherapy-driven effects, we focused our analysis on the contributions of ID3 and ID4, which have not been implicated in IL-6 regulation. To isolate chemotherapy-driven effects, we utilized OVCAR4 and OVCAR8 cell lines, which exhibited IL-6 induction in response to chemotherapy-associated ID upregulation but lacked baseline ID-dependent IL-6 expression. Consistent with our findings using AGX51 (Supplementary Figure S3C), siRNA-mediated knockdown of *ID3* or *ID4* did not significantly affect cell viability (Supplementary Figure S5C). To enable collection of conditioned media for analysis of IL-6 secretion following chemotherapy, cells were harvested on day 6 after siRNA transfection, corresponding to the waning phase of siRNA persistence [40]. At this waning stage, we observed a partial knockdown of *ID3* and *ID4* expression in response to carboplatin treatment (Supplementary Figure S5D,E). Notably, chemotherapy-enhanced IL-6 secretion was significantly reduced following knockdown of ID3 or ID4 in OVCAR8 cells and following ID4 knockdown in OVCAR4 cells (Supplementary Figure S5F). Taken together with the known roles of ID1 and ID2 on IL-6 expression, these data suggest that ID1-4 may contribute to IL-6 induction in response to chemotherapy.

In summary, these data indicate that ID proteins may drive IL-6 production in ovarian cancer cells in response to chemotherapy treatment, leading to a more permissive microenvironment for the survival of drug-resistant CSCs (Figure 6C).

## 4. Discussion

Our previous work demonstrated that 3D spheroid culture, which enriches for CSCs, induced upregulation of SMAD1/5/8 signaling and inhibitor of DNA-binding (ID) family transcription factors compared with 2D monolayer cultures, while also increasing tumor-initiating capacity and enhanced resistance to chemotherapy [6]. Consistent with this observation, ID protein expression is frequently induced downstream of BMP-mediated activation of SMAD1/5/8 signaling [41,42]. ID1-4 encode dominant-negative regulators of the bHLH family transcription factors, including E12, E47, and E2 [43], and have been linked to enhanced tumor cell proliferation, stem-like phenotypes, and increased metastatic potential in OC [12,13,14]. Previous studies in OC have largely focused on the roles of individual ID family members, describing roles for ID1 in angiogenesis, chemoresistance, and IL-6 signaling; for ID2 in E-cadherin regulation; for ID3 and ID4 in proliferation [19,41,44,45]. In contrast, genetic deletion studies in mice [9,10] and in spermatogonial stem cells using reporter and knockout models [46] demonstrate functional compensation among ID family members, although the compensatory mechanisms have not been examined in OC. In healthy tissue, ID protein expression is heavily regulated, remaining largely silenced until activated by extracellular cues such as TGF-β, BMP, IL-1β, and steroid hormone signaling, and subsequently limited by rapid ubiquitination and short protein half-lives [8,11,12]. In contrast, aberrant and sustained expression of ID1-4 has been reported in multiple cancer types and is frequently associated with aggressive disease and poor clinical outcomes [7,8]. Emerging evidence suggests that ID proteins are implicated in response to chemotherapy, where they may contribute to tumor cell survival [15,16]. These findings prompted us to investigate the role of ID proteins in CSC maintenance and CSC-driven remodeling during chemotherapy response.

To address this, we characterized the cumulative effect of ID proteins on CSC biology in ovarian cancer. We first leveraged publicly available datasets to determine whether coordinated ID expression represents an ovarian cancer-specific phenomenon or a broader feature of highly recurrent cancers. Analysis of TCGA datasets revealed that *ID1-3* are associated with malignancy and overall survival (Figure 1). Importantly, no single cancer type uniformly exhibited higher or lower ID expression; instead, heterogeneity was observed, with subsets of patients displaying either high or low ID expression. Furthermore, chemotherapy treatment increased *ID1-3* expression in OC patient samples and *ID1-4* in OC cell lines (Figure 2 and Figure 3). *ID1*, along with *ID2* and *ID3* expression in most OC cell lines, increased additively with repeated carboplatin exposures. In contrast, *ID4* often exhibited distinct expression trends, consistent with mounting evidence that *ID4* may play divergent roles in cancer biology [47]. In vivo, ID2 and ID4 returned to baseline following treatment cessation, indicating that chemotherapy-enriched ID expression is associated with drug-resistant cells and may influence IL-6 expression and the TME (Figure 4). Using a pharmacologic pan-ID inhibitor, we then interrogated how ID family activity supports CSC maintenance, EMT regulation, and TME remodeling. In OC cell lines, higher baseline ID expression was associated with greater sensitivity to pan-ID inhibition and partial abrogation of these CSC-associated features (Figure 5), while cell lines with lower baseline expression were less dependent on pan-ID inhibition. Functional pan-ID inhibition revealed that collective ID activity minimally affects CSCs, while strongly influencing IL-6 production (Figure 6).

To our knowledge, we are the first to examine the cumulative behavior of ID1-4 in response to chemotherapy treatment and assess how this influences CSC-associated features and microenvironmental interactions linked to relapse. Our findings suggest that cumulative ID activity represents a chemotherapy-enhanced adaptive program. Furthermore, this study represents the first evaluation of pan-ID inhibition using AGX51 in OC. AGX51 binds the loop region of ID proteins, inducing a conformational change that disrupts heterodimerization with bHLH binding partners and promotes N-terminal ubiquitination and proteasomal degradation via the 26S proteasome pathway [48]. One other study utilized ID1/ID3-specific aptamers to successfully induce cell death [49], supporting the relevance of ID1/3 inhibition and highlighting the need for simultaneous targeting of multiple ID family members. Importantly, we show that pharmacologic pan-ID inhibition selectively attenuates IL-6-associated TME signaling while only partially affecting CSC-associated features, supporting the feasibility of targeting ID-dependent adaptive responses during chemotherapy rather than attempting to eliminate CSCs directly.

CSC-driven tumor initiation capacity, chemoresistance, EMT regulation, and TME remodeling capabilities are governed by multiple overlapping pathways [1], including NF-κB, MAPK, Notch, Wnt, and TGF-β signaling, which may impact and compensate for ID activity. We have previously demonstrated that NF-κB signaling through both RelA and RelB supports CSC survival, proliferation, and self-renewal [6]. Additionally, we have established SOX2 as a key CSC marker in OC, where it plays a crucial role in maintaining stemness and promoting tumor growth, invasion, metastasis, and resistance to chemotherapy [5]. Previous studies have largely focused on individual ID family members in OC, implicating ID proteins in CSC maintenance, EMT regulation, and TME remodeling. ID1-mediated inhibition of E47 has been linked to increased cell death in vitro, reduced tumor growth in vivo, and adverse clinical outcomes, implicating ID1 in CSC maintenance [19,50]. ID1 also inhibits TCF12-SLC31A1 stabilization of the cisplatin influx transporter to increase chemoresistance [44]. RUNX1 can stimulate ID1- and ID3-dependent maintenance of CSCs, BCL2 expression, and inhibits CSC differentiation [51], while ID4 acts to support proliferation and differentiation by disrupting of HOX19 and CDKN1A [45]. ID proteins have also been implicated in EMT regulation, including ID2-mediated modulation of invasion through E-cadherin, regulated independently of the canonical HLH-binding domain [52]. Furthermore, ID expression both influences and is influenced by TME signaling factors that support CSCs, including the ID1–IL-6 axis in which IL-6 activates cell-intrinsic STAT3 and ATF6-dependent autophagy to promote resistance to chemotherapy-induced stress [27] and BMP4-mediated activation of ID3 [41]. ID1 can drive TME remodeling and induction of angiogenesis by activating AKT/NF-κB signaling in endothelial progenitor cells [53]. Collectively, this work suggests ID proteins are promising regulators of chemotherapy response and CSC-associated plasticity.

To further explore the CSC-associated ID response, we utilized an in vivo relapse model to examine the temporal dynamics of ID expression and found ID2 and ID4 expression were elevated in early response to chemotherapy. Analysis of residual tumors for CSC-regulating pathways like IL-6, STAT3, SOX2, and MAPK revealed a chemotherapy-associated increase in IL-6 expression, reduced caspase 8 cleavage, and sustained ERK, STAT3, SOX2, and cleaved RIP expression. Notably, ID2 and ID4 expression correlated with IL-6 levels, whereas only ID1 expression correlated with SOX2 expression. Because reduced caspase 8 cleavage has been implicated in impaired apoptotic signaling and poor clinical outcomes [54], and IL-6 has been identified through large-scale transcriptomic analysis as a driver of pro-tumoral, M2-like macrophage polarization in OC [33], we examined associations of IL-6 expression with markers of M1-like (CD80) and M2-like (CD163) macrophages. We observed a significant association between increasing IL-6 expression and loss of M1-like macrophages. Consistent with this, OC-associated IL-6 signaling has been linked to upregulation of PD-L1 on macrophages, contributing to immune evasion and tumor cell survival [55]. Furthermore, both chemotherapy-induced *IL6* gene expression and, more prominently, IL-6 secretion were significantly reduced following pan-ID inhibition. Using a related xenograft model in which ovarian cancer cells were injected intraperitoneally and collected either three days after chemotherapy or at tumor regrowth, we also observed decreased IL-23 and IL-12p70, M1-like cytokine expression [34]. Together, these findings support a role for ID proteins in chemotherapy-induced IL-6 that may contribute to TME remodeling, although additional studies are warranted.

Despite previous evidence implicating ID proteins in CSC maintenance and EMT regulation during chemotherapy, our data indicate that these pathways are only modestly affected by ID activity. Instead, IL-6 secretion may represent a more consistent response, whereas CSC maintenance appears to reflect heterogeneity within the ID family and differential patient responses. In support of this, OC cell lines with higher baseline ID expression exhibited partial dependence on ID signaling during chemotherapy, whereas cell lines with low baseline ID expression did not. Consistent with this interpretation, cells with low baseline ID expression also expressed high *SOX2*. These findings suggest multiple stress-responsive pathways activated by chemotherapy may converge to promote CSC-associated features. Interestingly, Meng et al. found that IL-6 secretion was linked to ID1 expression acting in an autocrine manner to activate STAT3 in signaling and promote chemoresistance and CSC features [27]; however, in our in vivo model, STAT3 activation remained largely unchanged following chemotherapy. In addition, exogenous IL-6 has been shown to increase ALDH activity in chemoresistant OC CSCs [28]. Although IL-6 stimulation in combination with chemotherapy did not increase spheroid formation or gene expression of *SOX2*, *NIK*, or *VIM*, stemness-associated factors identified previously [17], we observed increased ALDH activity and *SOX2* expression following carboplatin treatment, both of which were partially attenuated after pan-ID inhibition, in the OC cells with high baseline ID expression and chemotherapy-enhanced IL-6 expression. These findings highlight the complexity of chemotherapy-induced stress responses and underscore the need for future studies to dissect the intersecting pathways that govern CSC phenotypes during chemotherapy responses.

To further clarify the roles of ID family members in regulating IL-6 secretion in response to chemotherapy, we used siRNA-mediated knockdown of ID3 and ID4 and observed a significant reduction in chemotherapy-enriched IL-6 expression. To minimize confounding effects and isolate potential novel contributions to chemotherapy-enhanced IL-6 regulation, we focused on ID3 and ID4, which have not previously been linked to IL-6 signaling, whereas ID1 and ID2 have established associations with IL-6 regulation [27,39]. However, the roles of ID1 and ID2 in chemotherapy-induced IL-6 expression remain incompletely defined. Future studies will focus on identifying the molecular mechanisms by which ID family members regulate IL-6 expression in response to chemotherapy, including potential transcriptional, post-transcriptional, and stress-responsive signaling pathways like STAT3 or mTOR. In addition, evaluating the impact of ID inhibition during chemotherapy treatment in mouse models will be essential to determine how targeting cumulative ID activity influences IL-6-mediated tumor microenvironment remodeling and therapeutic response in vivo. Importantly, prior studies support the feasibility of AGX51-based combination therapies in ovarian cancer. Wojnarowicz et al. demonstrated that AGX51 in combination with paclitaxel significantly reduced breast cancer tumor progression in their xenograft murine model when compared with paclitaxel alone [35], providing in vivo evidence for the therapeutic potential of AGX51 in ovarian cancer.

Together, these data provide insight into ID1-4 dynamics in response to chemotherapy in highly recurrent cancers and highlight their potential contribution to chemoresistance and relapse. Further investigation into how ID proteins function in CSCs to regulate the ovarian TME may uncover novel drivers of tumor recurrence following chemotherapy.

## 5. Conclusions

In this study, we define a previously unappreciated collective role for the ID family in shaping the ovarian cancer response to chemotherapy. We demonstrate that ID1-4 are enriched following chemotherapy in both patient samples and experimental models, with expression increasing additively across treatment cycles. Functional pan-ID inhibition revealed that collective ID activity in response to chemotherapy only minimally affects CSC maintenance, while driving IL-6 production. Together, these findings highlight the importance of ID signaling in ovarian cancer cells to mediate pro-survival factors for tumor recurrence.

## Figures and Tables

**Table 1:** Gene Probes Information.

TaqMan Gene Expression Assay: *SOX2*	ThermoFisher	Hs01053049_s1
TaqMan Gene Expression Assay: *NOTCH3*	ThermoFisher	Hs01128537_m1
TaqMan Gene Expression Assay: *MMP2*	ThermoFisher	Hs01548727_m1
TaqMan Gene Expression Assay: *CDH2*	ThermoFisher	Hs00983056_m1
TaqMan Gene Expression Assay: *VIM*	ThermoFisher	Hs00185584_m1
TaqMan Gene Expression Assay: *ZEB1*	ThermoFisher	Hs01566408_m1
TaqMan Gene Expression Assay: *ID1*	ThermoFisher	Hs03676575_s1
TaqMan Gene Expression Assay: *ID2*	ThermoFisher	Hs00747379_m1
TaqMan Gene Expression Assay: *ID3*	ThermoFisher	Hs00171409_m1
TaqMan Gene Expression Assay: *ID4*	ThermoFisher	4331182-Hs02912975_g1
TaqMan Gene Expression Assay: *MAPK3K14 (NIK)*	ThermoFisher	4331182-Hs01089753_m1
TaqMan Gene Expression Assay: *GAPDH*	ThermoFisher	4448486-Hs02786624_g1

## Data Availability

Data presented in this study are available by request to the corresponding author.

## Supplementary Materials

The following supporting information can be downloaded at: https://www.mdpi.com/article/10.3390/cancers18081186/s1. Figure S1: Baseline *ID1-4* gene expression in ovarian cancer cell lines, Figure S2: IL-6 treatment of OC cell lines following chemotherapy treatment, Figure S3: Viability of OC cell lines following pan-ID inhibition with or without chemotherapy treatment, Figure S4: Pan-ID inhibition on CSC features following chemotherapy treatment, Figure S5: IL-6 baseline and *ID3* and *ID4* partial knockdown in OC cells reduces chemotherapy-enriched IL-6 secretion. File S1: Original western blots.
