# Supplementary material for: Chemotherapy Enrichment of ID Family Expression Is Associated with IL-6 Signaling in Ovarian Cancer
Source: Cancers (Basel). 2026 Apr 8;18(8):1186. doi: 10.3390/cancers18081186 (PMC13114274; doi:10.3390/cancers18081186)

0, 20, 40 uM AGX51 Blots

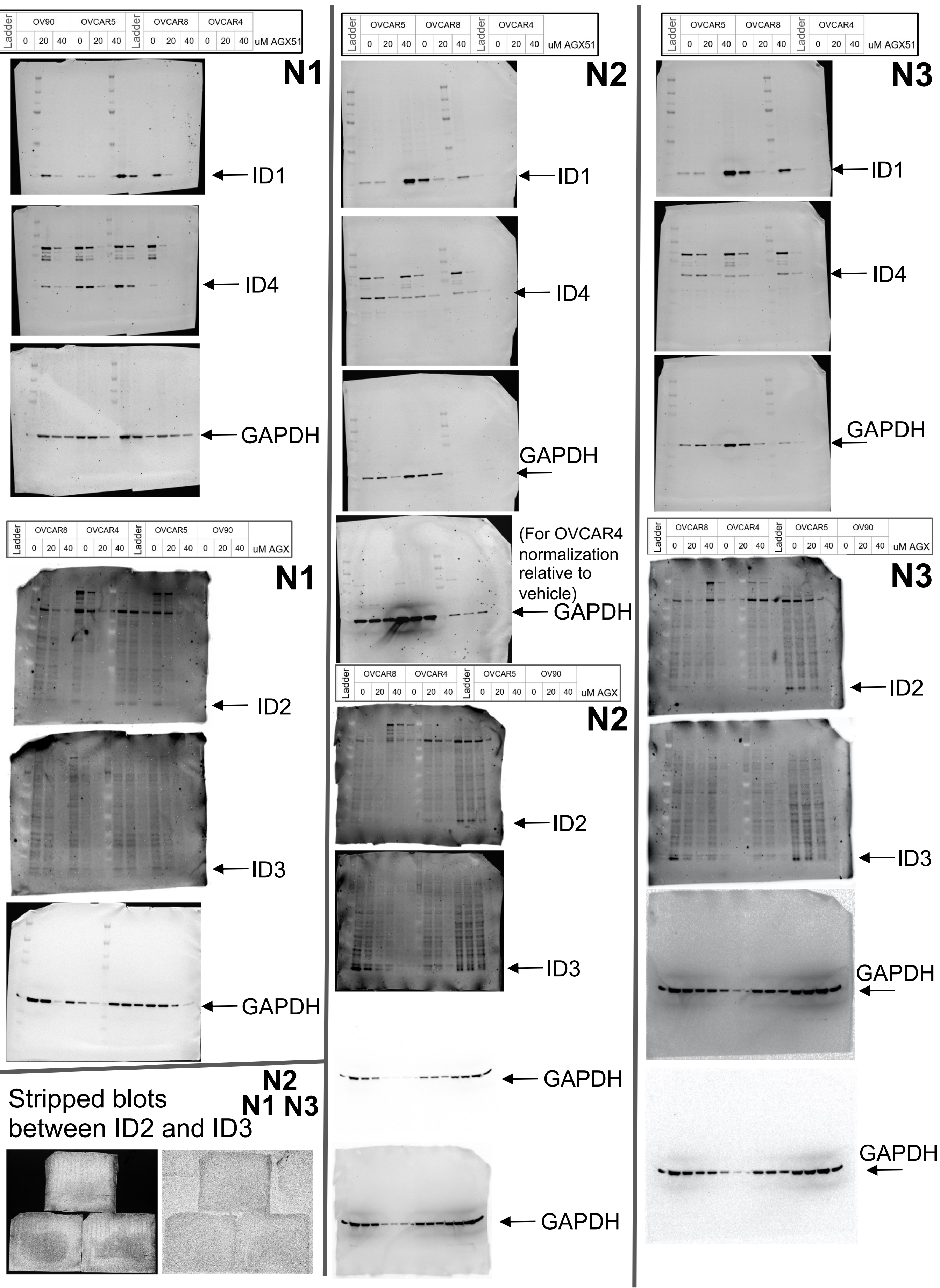

0, 20, 40 uM AGX51 Blots (cont'd)

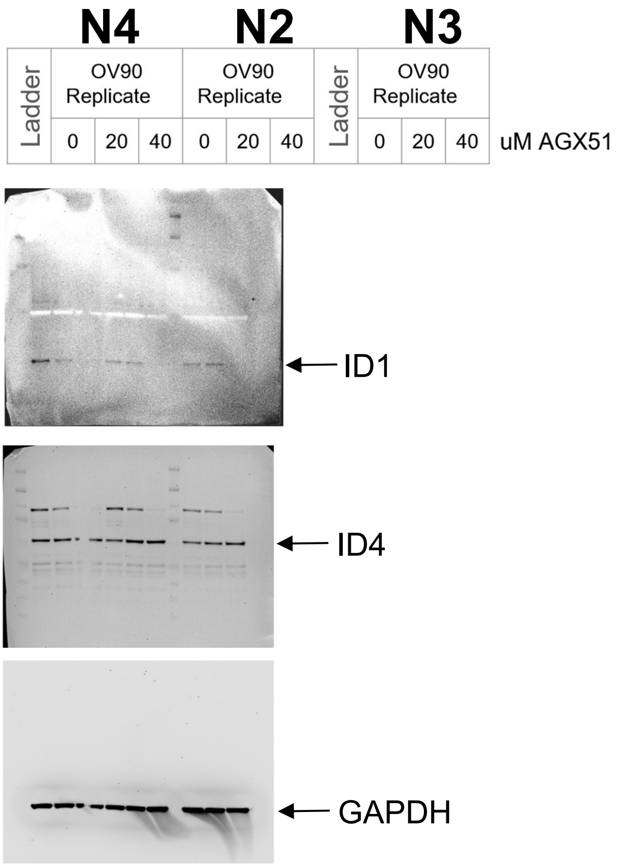

Vehicle vs Chemotherapy Blots

*V = Vehicle
*C = Chemotherapy treated

Lane	1	2	3	4	5	6	7	8	9	10	11
Sample	Ladder		OVCAR4		OVCAR5		OVCAR8		OV90		Ladder
			V	C	V	C	V	C	V	C	

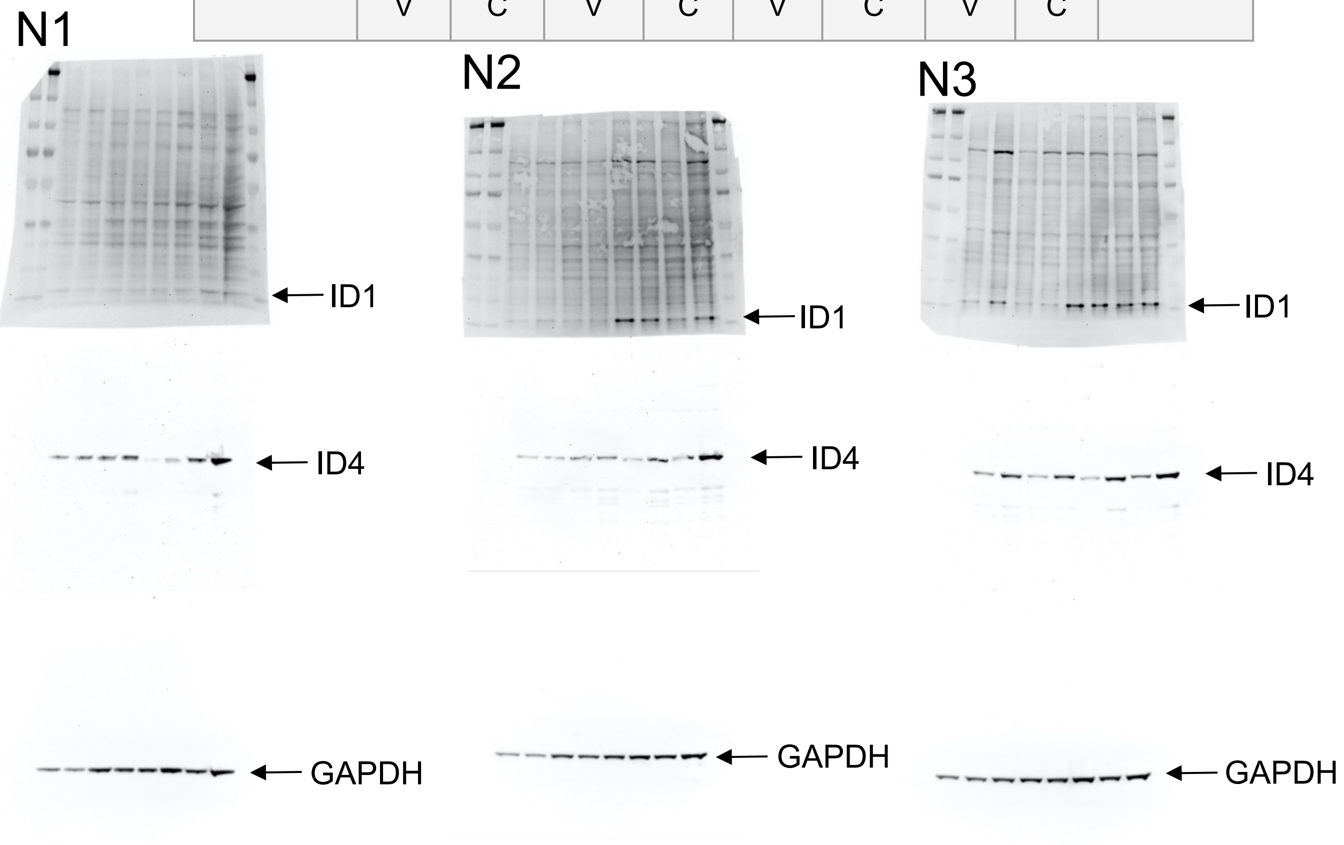

Lane	1	2	3	4	5	6	7	8	9	10	11	12
Cell Line	Empty	Ladder	OVCAR8		OVCAR4		Ladder	OVCAR5		OV90		Empty
treatment	-	-	vehicle	chemo	vehicle	chemo	-	vehicle	chemo	vehicle	chemo	-

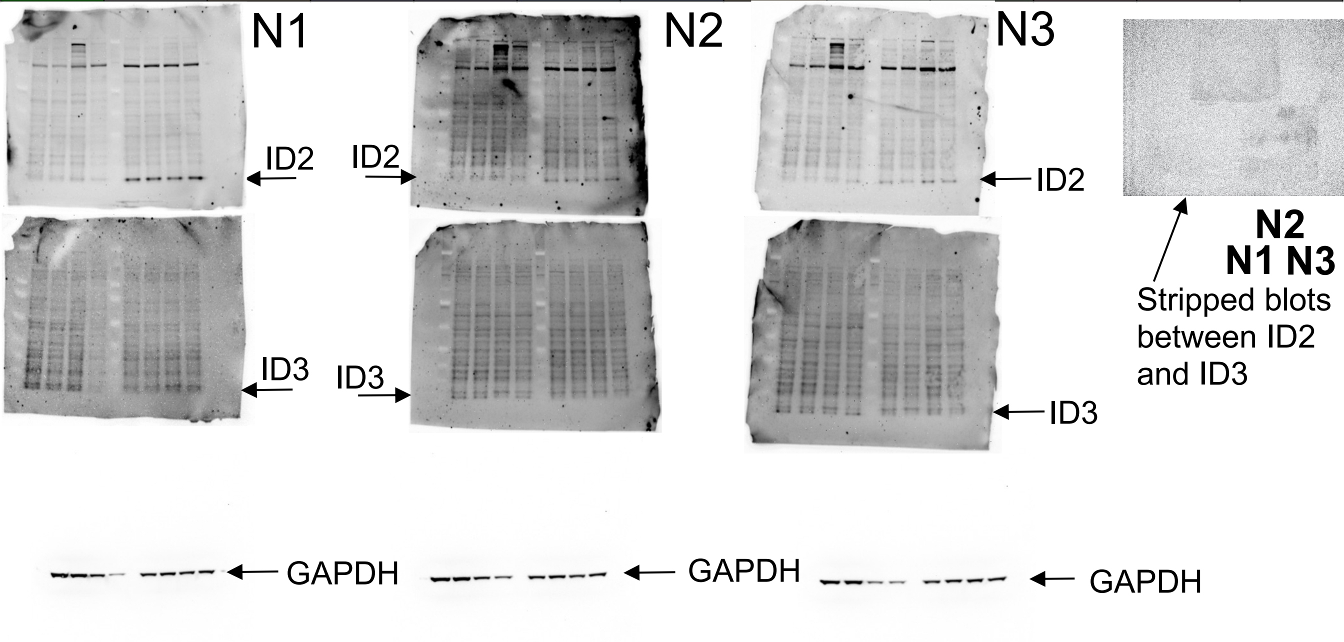

Supplement: Supplementary file 1 [file cancers-18-01186-s001.zip › cancers-4104285-File S1.pdf]
