# Supplementary material for: Chemotherapy Enrichment of ID Family Expression Is Associated with IL-6 Signaling in Ovarian Cancer
Source: Cancers (Basel). 2026 Apr 8;18(8):1186. doi: 10.3390/cancers18081186 (PMC13114274; doi:10.3390/cancers18081186)
Supplement: Supplementary file 1 [file cancers-18-01186-s001.zip › cancers-4104285-supplementary.pdf]

Supplementary Materials: Chemotherapy Enrichment of ID Family Expression is Associated with IL-6 Signaling in Ovarian Cancer

Megan Anne Keene, Darren Lighter, Cassandra Brenner, Ixchel Urbano, Katelyn Shelby, Samuel F Gilbert, Mikella Robinson and Carrie D House

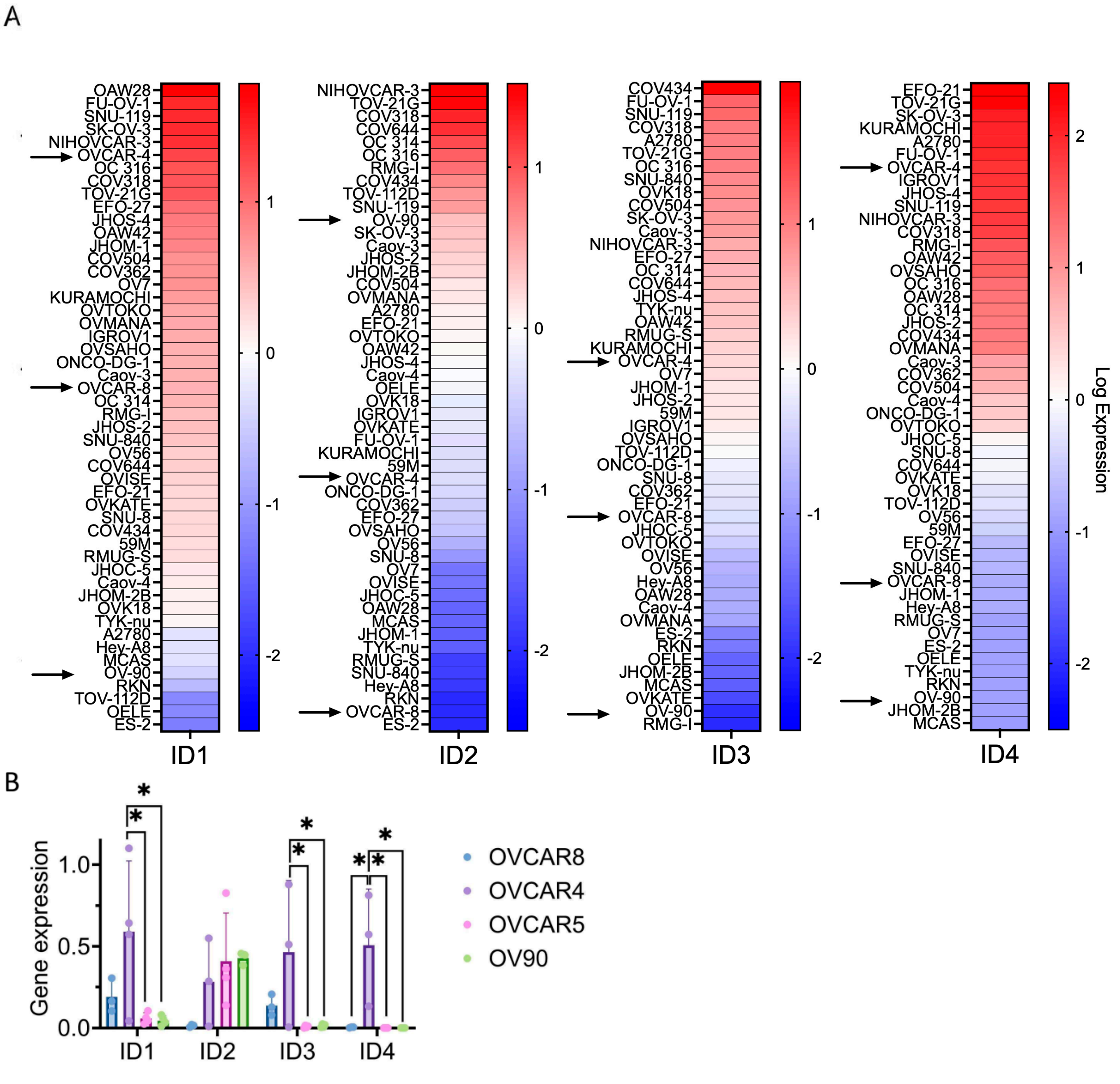

Figure S1. Baseline *ID1-4* gene expression in ovarian cancer cell lines A) Heatmap of Cancer Cell Lines Encyclopedia of *ID1*, *ID2*, *ID3*, and *ID4* gene expression (n=50). Arrows indicated cell lines used in this study. B) Baseline *ID1-4* gene expression in OVCAR8, OVCAR4, OVCAR5, and OV90 cells. n=3-4, one-way ANOVA, Tukey post-hoc test. * $p<0.05$.

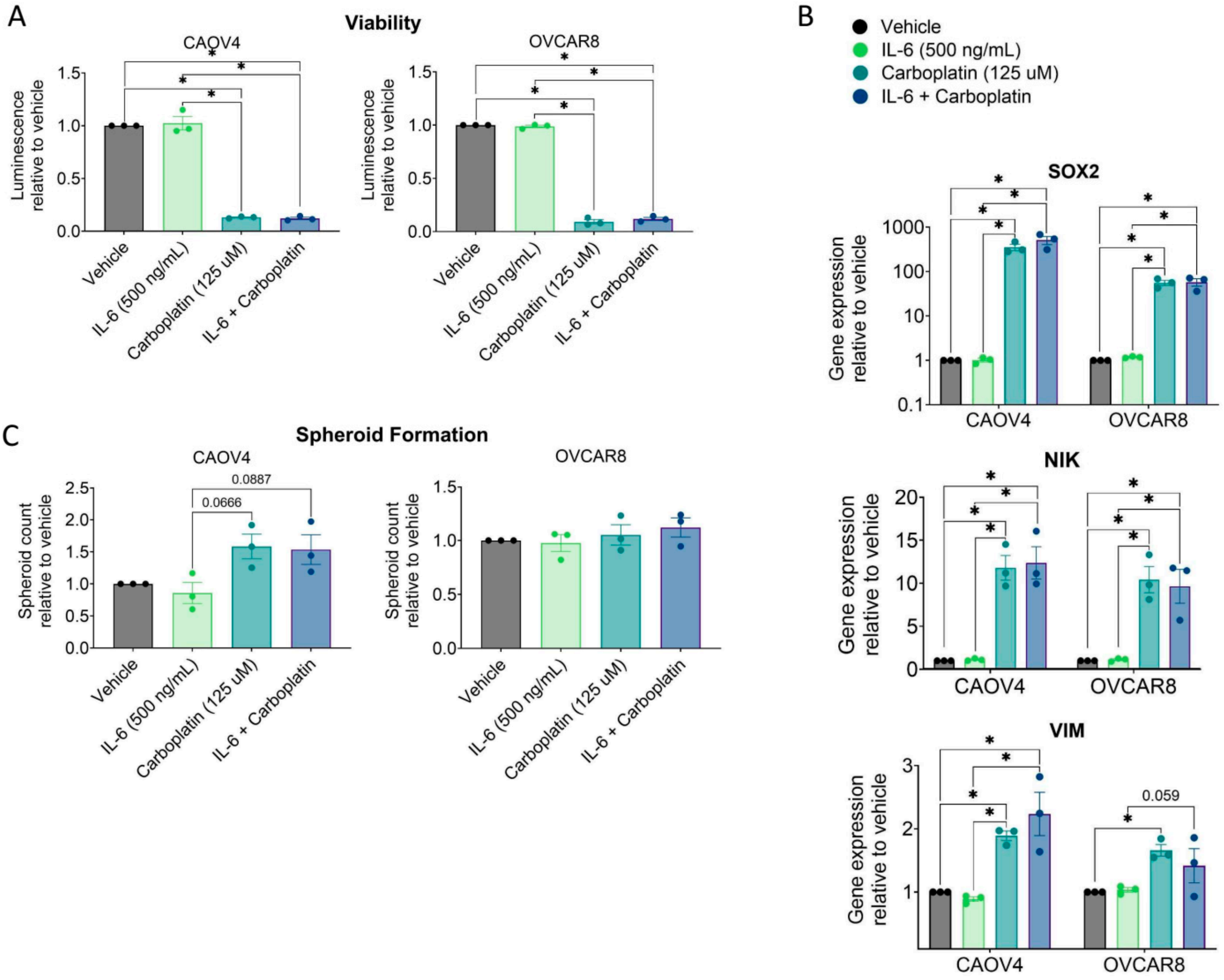

Figure S2. IL-6 stimulation of OC cell lines during chemotherapy treatment A) Viability of CAOV4 and OVCAR8 cells treated with vehicle, IL-6 (500 ng/mL), and/or carboplatin (125 µM) for 72 hours. n=3, one-way ANOVA, Tukey post-hoc test. B) *SOX2*, *NIK*, and *VIM* gene expression of CAOV4 and OVCAR8 cells treated with treated with vehicle, IL-6 (500 ng/mL), and/or carboplatin (125 µM) for 72 hours. n=3, one-way ANOVA, Tukey post-hoc test. C) Relative spheroid formation of CAOV4 and OVCAR8 cells treated with vehicle, IL-6 (500 ng/mL), and/or carboplatin (125 µM) for 4 days. n=3, one-way ANOVA, Tukey post-hoc test. * $p<0.05$.

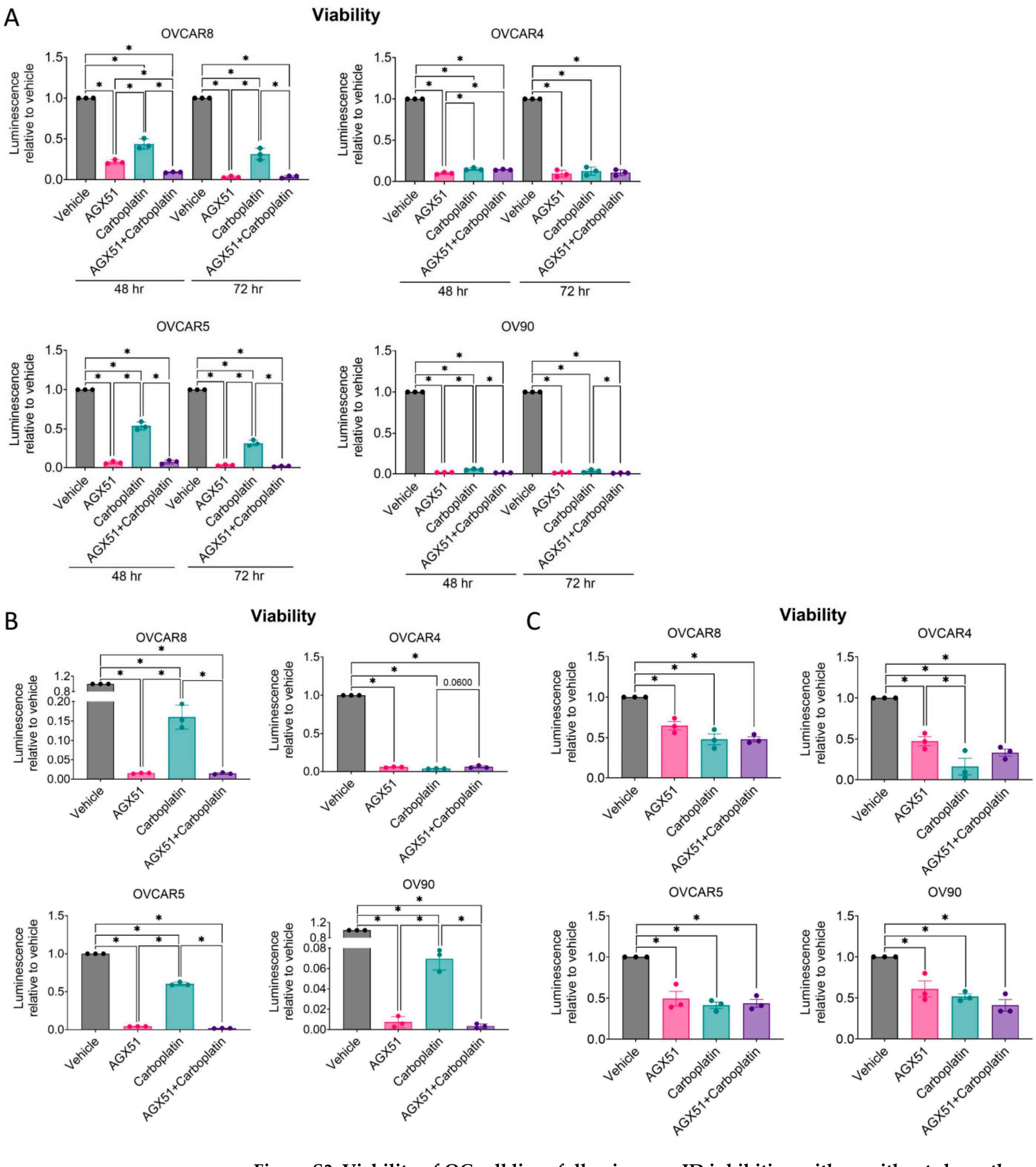

Figure S3. Viability of OC cell lines following pan-ID inhibition with or without chemotherapy treatment A) Relative viability of OVCAR8, OVCAR4, OVCAR5, and OV90 cells treated with vehicle, 40 µM AGX51, and/or 100 µM carboplatin (40 µM for OVCAR8) for 48 or 72 hours. n=3, one-way ANOVA, Tukey post-hoc test. B) Relative viability of OVCAR8, OVCAR4, OVCAR5, and OV90 cells with 24 hours pretreatment of 40 µM AGX51, followed by drug washout and 72 hours treatment of 100 µM carboplatin (40 µM for OVCAR8). n=3, one-way ANOVA, Tukey post-hoc test. C) Relative viability of OVCAR8, OVCAR4, OVCAR5, and OV90 cells with 24 hours pretreatment of 20 µM AGX51, then continued concurrently with vehicle or 100 µM carboplatin (40 µM for OVCAR8) treatment for 72 hours. n=3, one-way ANOVA, Tukey post-hoc test. * $p<0.05$.

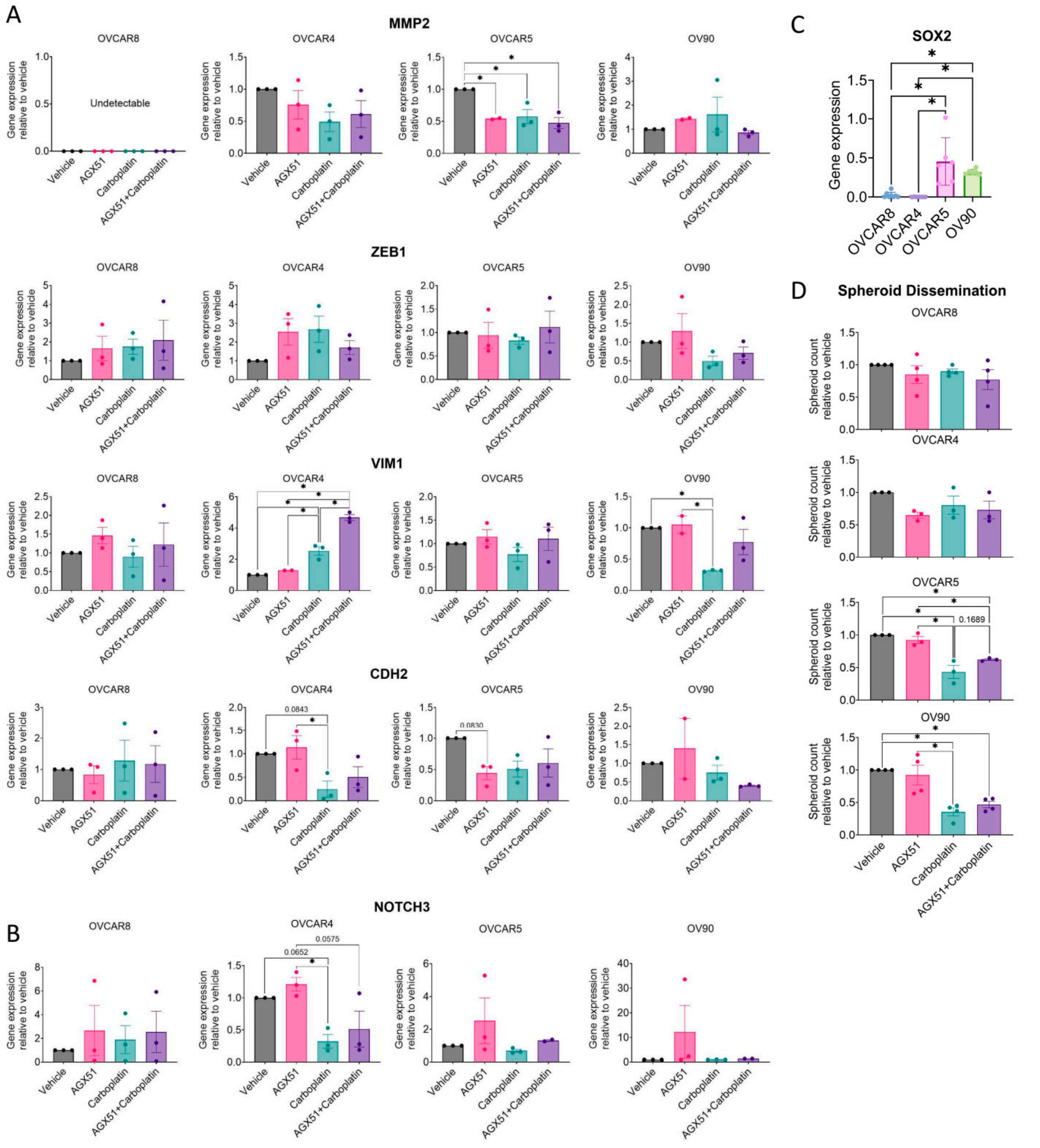

Figure S4. Pan-ID inhibition on CSC features following chemotherapy treatment A) Relative *MMP2*, *ZEB1*, *VIM1*, and *CDH2* gene expression in OVCAR8, OVCAR4, OVCAR5, and OV90 cells pretreated with vehicle or 20 μM AGX51 for 24 hours, then continued concurrently with vehicle or 100 μM carboplatin (40 μM for OVCAR8) treatment for 72 hours. n=3, one-way ANOVA, Tukey post-hoc test. B) Relative NOTCH3 gene expression in OVCAR8, OVCAR4, OVCAR5, and OV90 cells pretreated with vehicle or 20 μM AGX51 for 24 hours with 24 hours pretreatment of 20 μM AGX51, then continued concurrently with vehicle or 100 μM carboplatin (40 μM for OVCAR8) treatment for 72 hours. n=3, one-way ANOVA, Tukey post-hoc test. C) Baseline *SOX2* gene expression in OVCAR8, OVCAR4, OVCAR5, and OV90 cells. n=6-8, one-way ANOVA, Tukey post-hoc test. D) Relative spheroid dissemination of OVCAR8, OVCAR4, OVCAR5, and OV90 cells. Spheroids were grown for 4 days then treated with 20 μM AGX51 and/or 100 μM carboplatin (40 μM for OVCAR8) for 4 days. n=3-4, one-way ANOVA, Tukey post-hoc test. * $p<0.05$.

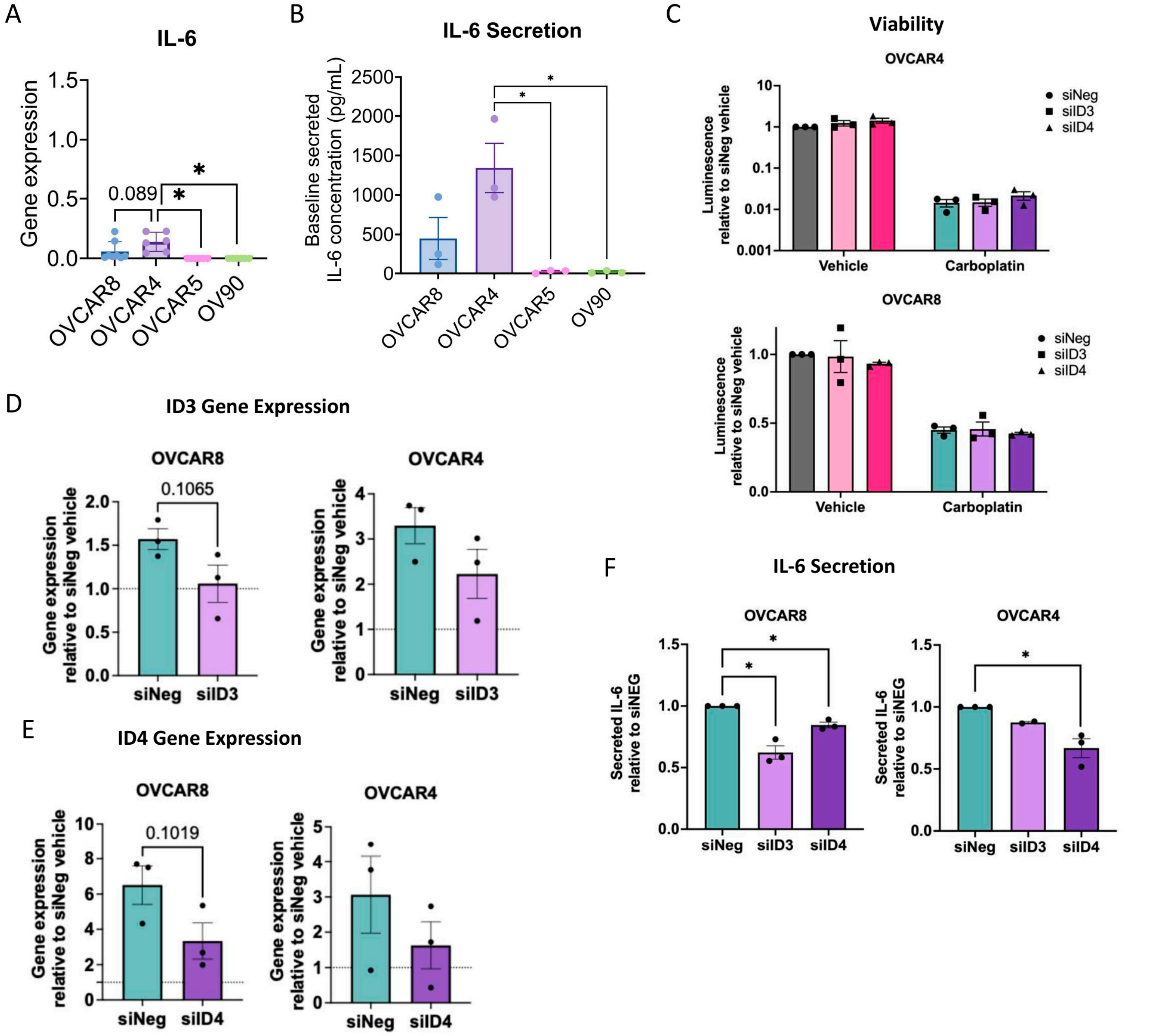

Figure S5. IL-6 baseline and *ID3* and *ID4* partial knockdown in OC cells reduces chemotherapy-enriched IL-6 secretion A) Baseline *IL6* gene expression in OVCAR8, OVCAR4, OVCAR5, and OV90 cells. n=6-8, one-way ANOVA, Tukey post-hoc test. B) Baseline secretion of IL-6 in conditioned media from OVCAR8, OVCAR4, OVCAR5, and OV90 cells. n=3, one-way ANOVA, Tukey post-hoc test. C) Viability of OVCAR4 and OVCAR8 cells after siRNA knockdown of scramble (neg), *ID3* or *ID4*, followed by treated with vehicle or carboplatin (100 or 40 μM, respectively) for 72 hours. n=3, one-way ANOVA, Tukey post-hoc test between siNeg, siID3, and siID4. D-E) Gene expression of *ID3* (D) and *ID4* (E) for OVCAR4 and OVCAR8 cells after siRNA knockdown of scramble (neg), *ID3* or *ID4*, followed by treated with carboplatin (100 or 40 μM, respectively) for 72 hours. Gene expression is normalized to siNeg vehicle and shown as fold change of carboplatin-treated samples relative to their respective vehicle controls. N=3, unpaired t-test. F) Secretion of IL-6 in conditioned media from OVCAR4 and OVCAR8 cells after siRNA knockdown of scramble (neg), ID3 or ID4, followed by treatment with carboplatin (100 or 40 μM, respectively) for 72 hours. N=3, one-way ANOVA, Dunnet post-hoc test to siNeg treatment. * $p<0.05$.
